# Qualitative investigation of patient and carer experiences of everyday legal needs towards end of life

**DOI:** 10.1186/s12904-021-00739-w

**Published:** 2021-03-23

**Authors:** Helen Close, Kamal Sidhu, Hazel Genn, Jonathan Ling, Colette Hawkins

**Affiliations:** 1 Institute of Health and Society, Baddiley-Clark Building, Faculty of Medical Sciences, Newcastle University, Newcastle upon Tyne, NE2 4AX UK; 2 Blackhall and Peterlee Practice, Hesleden Road, Blackhall Colliery, County Durham, TS27 4LQ UK; 3 UCL Centre for Access to Justice, UCL Faculty of Laws, Bentham House, 4-8 Endsleigh Gardens, London, WC1H 0EG UK; 4 Public Health, Faculty of Health Sciences and Wellbeing, Sunderland University, Sunderland, SR1 3SD UK; 5 Palliative Medicine, St Oswald’s Hospice, Regent Avenue, Gosforth, Newcastle upon Tyne, NE3 1EE UK; 6 Present address: Palliative Medicine, Newcastle upon Tyne Hospitals NHS Foundation Trust, Freeman Hospital, Freeman Road, Newcastle upon Tyne, NE7 7DN UK

**Keywords:** Holistic care, social welfare, legal rights, end of life, advance planning, palliative care

## Abstract

**Background:**

Legal issues are common in chronic illness. These include matters of daily life, such as problems with employment, finances and housing, where rights or entitlements are prescribed by law. They also include planning ahead, for example, making a Lasting Power of Attorney. However, the nature, impact and management of legal needs in the context of end of life care are not known. This study investigated these from the perspectives of patients and carers.

**Methods:**

Patients, with estimated prognosis 12 months or less, and carers were recruited from two sites: day services within an urban hospice and primary care in an area of deprivation in North-East England. Semi-structured interviews explored the nature and impact of legal issues, access to appropriate support and unmet needs. Thematic analysis of data was undertaken.

**Results:**

Twenty-seven interviews were conducted with 14 patients (10/14 hospice) and 13 carers (7/13 hospice). Five were patient-carer dyads. All participants had experienced problems raising legal issues, which generated significant practical and psychological challenges. All had struggled to access support for social welfare legal issues, describing not knowing what, who, or when to ask for help. All participants accessed some support, however routes, timing and issues addressed were variable. Facilitators included serendipitous triggers and informed healthcare professionals who offered support directly, or signposted elsewhere. A range of professionals and organisations provided support; resolution of issues conferred substantial benefit. The majority of participants identified unresolved legal issues, predominantly related to planning ahead. The challenge of facing increased dependency and death proved a key barrier to this; informed and compassionate healthcare professionals were important enablers.

**Conclusion:**

Everyday legal needs are a common and distressing consequence of life-limiting illness, affecting patients and carers alike. This study identified inconsistent approaches but practical and psychological benefit when needs were met. Healthcare professionals were central to meeting social welfare legal needs and facilitating effective planning, with important roles as ‘critical noticers’, trusted intermediaries and compassionate communicators. Increased awareness, clearer pathways to support and closer service integration are needed to meet legal needs as a component of holistic care.

## Background

Life-limiting illness generates wide-ranging challenges for patients and their carers. A holistic approach to assessment and management is a core philosophy within palliative care [1], and a system-wide response, using a ‘full range of co-ordinated services’ is advocated for people with an estimated prognosis less than one year [2].

Problems raising everyday legal needs are very common in chronic illness [3]. They relate to social welfare matters for which the law defines rights, entitlements and protections, including income security, suitable housing, employment rights, family issues, immigration, protection from abuse and the right to community care [3, 4]. These social welfare legal (SWL) needs tend to cluster, disproportionately affecting people with multiple health needs, mental illness and/or social disadvantage [4]. Evidence shows significant unmet need for advice and assistance with SWL problems in the context of ill health, thought to relate to a combination of factors, including lack of awareness of rights and no clear pathway to assessment and support [5, 6]. Unmet legal needs become chronic stressors, generating morbidity in their own right [7, 8] and exacerbating chronic ill health [3, 8, 9]. The extent to which these findings apply to people living towards end of life and their carers is not known.

Healthcare workers, particularly General Practitioners (GPs), are often the first point of contact for legal needs with a social welfare basis [3, 10]. As well as adding strain on GP services, the majority of GPs need specialist input to manage these issues with most signposting onto external advice services [10]. This highlights the role that healthcare professionals play as ‘critical noticers’ of SWL needs and their value as trusted intermediaries in facilitating access to support [3].

SWL support is provided by a range of services across health and social care, hospices, councils, advice sector, private and charitable legal services and a range of health-related charities. These often operate independently of each other, with no consistency in language or approach to legal needs [11]. Variability in practice risks inequitable care and unmet needs [11].

Partnerships between health and SWL advice services do already exist. In Australia and USA, there is a co-ordinated effort towards a national network of integrated services [12, 13]. Legal and SWL advice services are considered key enablers of effective healthcare and evidence is beginning to demonstrate the benefit of partnerships on accessibility and a wide range of outcomes [3, 10–13]. In England and Wales, over 380 services operate a referral pathway from healthcare into a SWL advice service; most are locally defined and almost 40% funded for less than one year [14].

As well as the challenges of daily life, life-limiting illness encourages consideration of advance planning, including choices around medical treatment and care, funeral, organising property, finances and guardianship of dependents. These have legal implications either because they are legally binding in themselves or because they are associated with rights described by the Mental Capacity Act 2015 [15] or the Human Rights Act 1998 [16].

Autonomy is recognised as a basic human right, generating a responsibility on care providers to facilitate individual autonomy, including choices around care towards end of life [17, 18]. This is particularly challenging in the context of advance care planning since the topic itself can be distressing and choices relate to hypothetical situations. A re-framing of individual autonomy as relational autonomy, with decisions made by individuals together with significant others, has been proposed [19].

The aim of this project was explore the question: ‘What is the experience of everyday legal needs amongst people approaching end of life and their informal carers?’ Objectives were to investigate the nature and impact of everyday legal needs, routes to support and the extent of unmet need.

## Methods

This project focused on developing our understanding of how people experience legal needs at the end of life. We used reflexive thematic analysis with an inductive approach [20], with coding and theme development directed by interview data without a pre-existing coding frame.

Everyday legal needs were defined as:

- Current social welfare issues associated with rights and entitlements: finances/benefits (including insurance/pension payments), employment, housing (ownership, adaptations and aids), social care, driving (responsibilities and disability rights).
- Future planning with legal rights or protections: will, Lasting Power of Attorney, advance care planning, funeral planning/prepayment.

### Study population and sample strategy

Patients and carers were recruited from two sites: a day service within a hospice situated in an affluent urban area and providing care for patients and their carers across the North East of England, and a large two-site GP practice situated in a highly deprived area of North East England serving a mixed urban/rural area. The sites were selected as they offered a cross section of socio-economic groups and both primary and specialist care. The hospice was selected as a specialist regional unit; none of the investigators had prior working relationships with this site. The primary care site was identified through regional networking; KS, clinical lead at Blackhall and Peterlee, agreed to support patient/carer recruitment. Patient records were screened by staff at each site (GP practice manager and hospice outpatient staff) to identify patients meeting eligibility criteria. Patients and main carers were then purposively sampled by the researchers (from the same and different family units) and approached by clinical staff to participate in a 30-45 minute individual interview. Purposive sampling was used in order to recruit people with a range of conditions, both malignant and non-malignant (see Table 1), as support structures vary according to diagnosis.

**Table 1:** Description of interviewees

	No of interviews	Recruitment site	Gender	Mean Age (range)	Diagnoses
**Patients**	14	10 (71%) hospice 4 primary care	6 (43%) male	70.3 years (38-87)	10 (71%) cancer; 2 COPD; 1 Multiple System Atrophy; 1 Muscular Dystrophy
**Carers**	13	7 hospice 6 primary care	7 (54%) male	67.8 years (46-82)	5 (38%) cancer; 3 Progressive Supranuclear Palsy; 1 Multiple System Atrophy; 1 Motor Neurone Disease; 2 dementia; 1 COPD

Recruitment ended when repeated themes had been identified and the researchers felt able to present the issues which mattered most to the participants. Eligibility criteria included age over 18 years, ability to consent, a history of any life-limiting illness (apart from severe mental illness) with an answer to the ‘surprise’ question (“Would you be surprised if this person dies within the next 12 months?” [21]) of ‘no’ as judged by their clinical link person.

### Procedure

Interviews took place at either the hospice day unit or in the patient/carer’s own home according to preference. HC conducted all interviews. A semi-structured interview guide was used, developed by a multi-professional project steering group which included patient/carer representatives. The guide incorporated questions about current experience and plans for the future. The context of the interview was described both within the participant information sheet and at the beginning of the interview. Everyday legal needs were introduced broadly as including “*issues around money and benefits, employment, housing, or planning for the future*”. Additional questions allowed participants to expand on other relevant or related concerns and more specific questions, such as experience around Power of Attorney, were included where appropriate, to capture breadth of legal need. Interviews were audio recorded and transcribed verbatim. After every 2-3 interviews, the transcribed interviews were reviewed by the interviewer and changes made to the interview schedule as part of an iterative process. Separate interviews for patients and carers within dyads were planned, although in three cases patients and carers were interviewed together.

### Data analysis

Data analysis was led by HC using the six-phase approach to reflexive thematic analysis [20, 22], to explore the lived experience of participants. This entailed familiarization with the data then initial coding through application of descriptive words to significant phrases. This list of codes was captured using a Microsoft Excel spreadsheet with examples of quotes taken from individual transcripts. Codes were grouped together into meaningful themes. Themes were reviewed, refined and tested against the dataset through in-depth discussion (HC and CH), as part of the reflexive approach, ensuring themes reflected the data and aligned with the research question. Both researchers have clinical experience; CH as a consultant in palliative medicine, HC as a former District Nurse with specialist palliative care experience, now an experienced qualitative researcher. Standards for Reporting Qualitative Research guidelines were used in manuscript preparation [23].

**Fig. 1:**
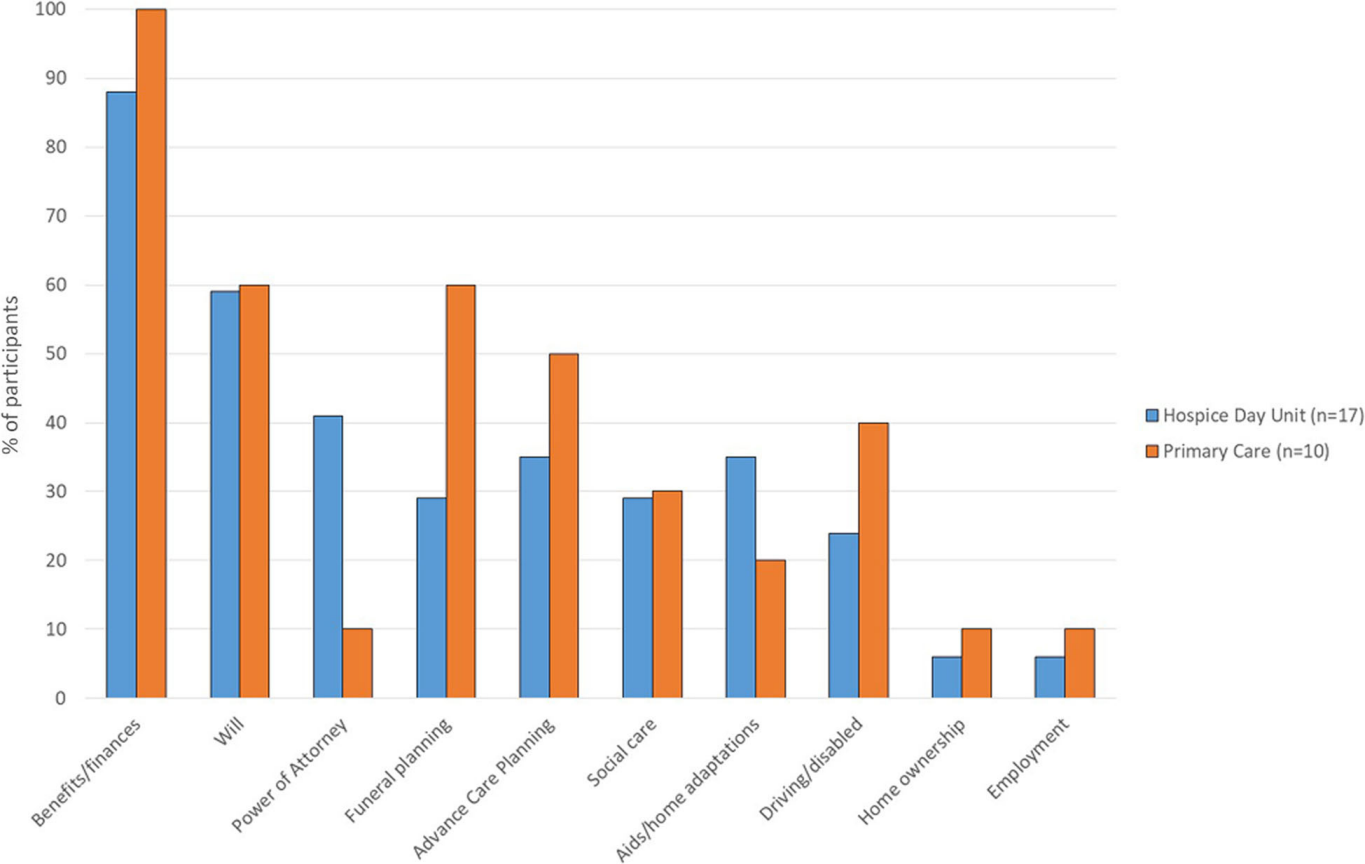
Legal Needs Addressed

**Fig. 2:**
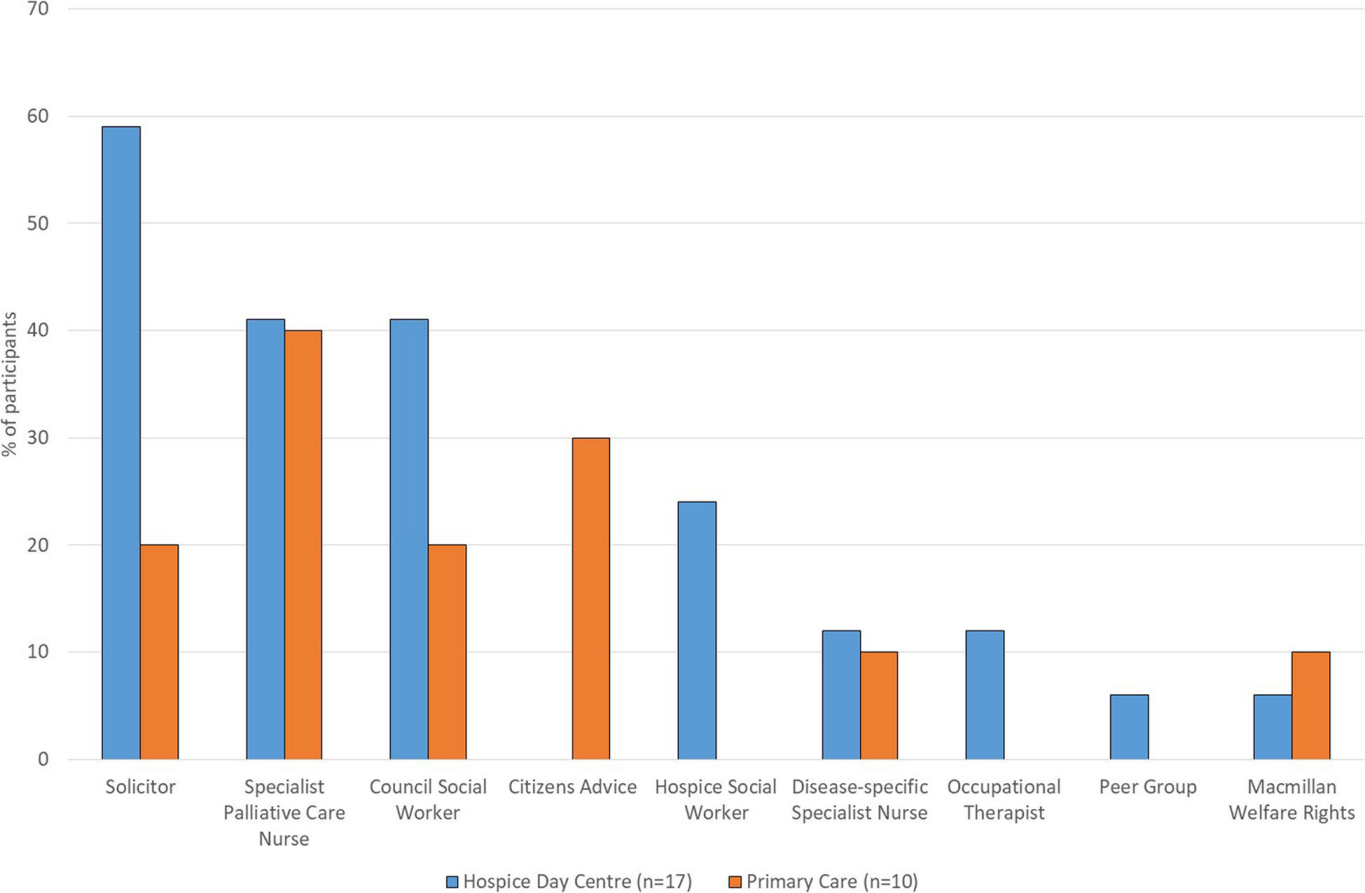
Sources of Professional Support for Social Welfare Legal Needs

**Fig. 3:**
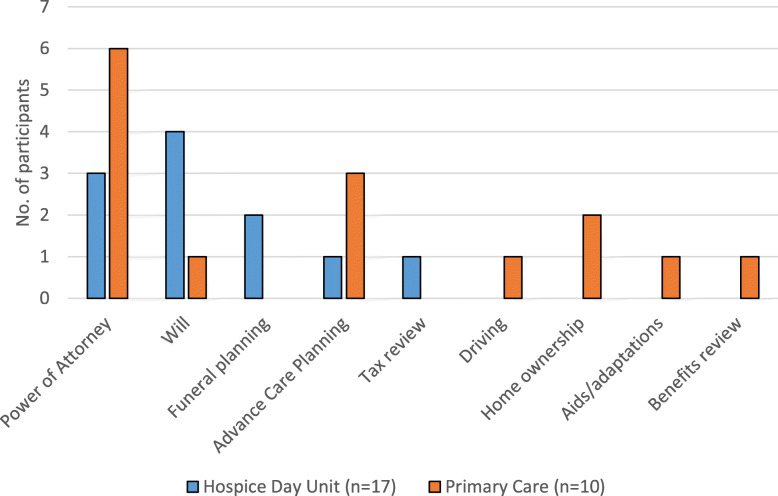
Outstanding Legal Needs, Support Wanted

As we were interested in finding out whether there were any differences in legal needs and routes to support between patients in the two settings, we grouped hospice participants and primary care participants separately when analysing this data (Figs 1, 2 and 3). Participants were allocated identifier codes (HP = hospice patient, HC = hospice carer, PCP = primary care patient, PCC = primary care carer) with consecutive numbers.

### Ethical considerations

Research ethics approval was granted by East Midlands (Nottingham 1) NHS Ethics Committee (REC: 18/EM/0021, February 2018). Health Research Authority approval was granted (IRAS ID 236775). Research was conducted in accordance with the Declaration of Helsinki. Potential participants were given both verbal and written information about the project and implications for participation. Staff at both recruitment sites were educated about the project, in recognition that patients and carers may direct questions to familiar professionals; contact with project team members was also available. A week interval between invitation to participate and contact to book an interview allowed time for questions and consideration. Written informed consent was obtained from participants in the study immediately prior to interview. Audio and written data was pseudonymised and stored according to the sponsor’s information governance regulations and the Data Protection Act 1998. Participants were allowed to remove their data from the study prior to anonymization, however none elected to do so. Active concerns identified during the interview were further discussed following the interview with the offer to refer onto appropriate services.

## Results

### Participant demographics

A total of 27 interviews were conducted with 14 patients with an estimated prognosis of less than 12 months and 13 carers (Table 1). Three patient/carer dyads were recruited from the GP practice and requested to be interviewed together; two patient/carer dyads were recruited from the hospice but were interviewed separately.

Time since diagnosis varied from 1 year to 15 years. The majority of carers (11/13) were spouses of patients with prognosis less than 12 months, two were adult children. Five of the carers were linked to people living in care homes. Five patients lived alone, 1 lived in a care home.

### Experience of everyday legal needs

Interviews captured participant experience of everyday legal needs within the disease journey, both in terms of those already addressed (Fig. 1) and outstanding issues. All participants had experience of legal needs resulting from life-limiting illness, relating to both current SWL needs and future planning. The nature of everyday legal needs appeared broadly similar between the two recruiting sites and between patients and carers.

SWL needs were addressed by direct management and/or signposting by professionals. This support came from a wide variety of face-to-face services (Fig. 2).

Some participants recalled a healthcare professional who had signposted them on to SWL support. These included their GP (4 participants), District Nurse (1), Specialist Nurse (2), physiotherapist (1) and chemotherapy nurse (1).

Six participants, all hospice recruits, felt well organised and supported, with no outstanding legal needs identified at interview. All described sources of support including dynamic and informed professionals and/or informed family or friends.

The remaining participants (11 hospice, 10 primary care) all identified unresolved legal needs during the interview. Most (8 hospice, 7 primary care) wanted help to resolve these (Fig. 3). For hospice participants, all but one related to planning ahead; for primary care participants there was a mix of current needs and planning ahead.

Participants seeking support were either referred to appropriate services following the interview, or stated their intention to self-refer to a trusted source of support.

In addition, seven hospice recruits and five primary care recruits identified unmet everyday legal needs but wanted to leave them unaddressed. These all related to planning ahead: Lasting Power of Attorney (6), wills (5) and advance care planning (6). The main reason behind this was the challenge of facing deterioration and death and conversations around this. Other reasons reported were difficulty identifying an attorney (1), family member discouraging planning (1), perceived cost (1), and little money to manage/leave for an inheritance (2).

### Main themes

Interviews revealed a common story around the impact of everyday legal needs on participants’ lives, the challenges and routes to accessing support, the timing of support and the impact this support had on them. We identified differences between SWL needs and planning in advance, in relation to accessing support, so these are presented separately.

#### Theme 1: The impact of active everyday legal needs

Most participants described psychological impacts of active everyday legal needs (anxiety, depression, difficulty sleeping), usually at a time they were already struggling with ill-health. This affected patients and carers alike. Some felt overwhelmed.

‘*Because at the time …, I was worried about everything and I was starting to get upset and anxious about it all. It is just you don’t know what you are eligible for. How are you meant to know? Making sense of the information … and how it relates to you is virtually impossible.’ (HP11)*

*‘Yeah more information about your rights and things would be great.., yes, because you don’t know who to turn to when you first, you know, get the word of what it is and you think oh crikey what on earth am I going to do? What do I do?’ (HC4)*

#### Theme 2: Challenges to accessing support for SWL needs

##### Finding information and support

Participants had limited understanding of how to access SWL advice and support. Without exception, patients and carers felt they did not know what they should be asking for, who to ask, or when to ask.

*‘I didn’t know who to go to, to be honest with you, I didn’t know what to do, so I just muddled on the best way I could, you know. ... I didn’t know if I could get help…nobody was coming forward with any. I just didn’t know where to go.’ (PCP2)*

*‘So you know the frustration is massive when you don’t know what to ask for… it’s not just the information, it’s also where to ask, who do you contact when you settle on those things that are available, how do you make those connections about what you need, how do you write the right letter and also trying to get help and how do you get follow-up?’ (HC7).*

Even those with degree-level education, internet access and IT skills struggled to navigate the complexity and volume of information available online.

*‘I tried to look for the PIP [Personal Independence Payment]. Somebody had said to me that you’ll be able to claim PIP, so I had a look on a Government website for PIP. I just didn’t know, I couldn’t make head nor tail of it. It just seemed too much information for me…’ (HP11)*

This resulted in situations where people either waited for months or years to be offered the right support, or they tried to navigate the systems themselves, often unsuccessfully. This was identified as a source of frustration as well as generating significant hardship.

*‘Yeah, but it [PIP] took about a year all told. It was a long time I know. And, well, I was living from hand to mouth then.’ (PCP4)*

*‘It took two and a half years to crack [get support from] the [benefits] system’ (HC7).*

##### Assumptions and beliefs about eligibility

Some patients and carers wrongly assumed that their previous working status would prevent them from being eligible for help - financial or otherwise - and this was a barrier to asking for help.

*‘Well, because you’ve got your old age pension and you’ve got your private pension, you think you’re not entitled to anything.’ (PCP4)*

*‘I’ve always worked so I says I’m not entitled to nothing but I was wrong.’ (PCP1)*

For some, this was compounded by a sense of pride in one’s own ability to be self-sufficient, together with a sense of shame at having to rely on external resources.

*‘I don’t want anything from the Government for which I’m not allowed. I don’t want to be a burden on them.’ (HP5)*

*‘The first thing I told her [specialist palliative care nurse] when she was here was that I had a wonderful Coal Board pension, who can keep me in the sort of, not exactly luxury, but it keeps me with everything that I need until the time when I don’t need it.’ (PCP3)*

##### Fight for rights

The majority of patient and carers expressed frustration at the sense of having to act as one’s own advocate and having to pull legal and welfare information together oneself. When describing the process of navigating services and support, strong language and analogies of war were used by patients and carers alike*.*

*‘… the situation puts you in a position that you must know everything. And also you … have to advocate for it, and fight for it and then the information is given to you.’ (HC7)*

*‘I finally got me CHC funding after a battle.’ (HP10)*

This perceived fight for rights was an unwanted extra burden for patients and carers who were already struggling to cope with progressive ill health. It had clear consequences for the quality of life of patients and the physical and mental health of carers.

*‘They [SWL needs] leave you disabled and struggling to manage day to day. They distract from what’s important.’ (HC7)*

#### Theme 3: Routes to support for SWL needs

##### Serendipitous factors

The majority of participants reported an apparently unstructured approach to addressing SWL needs. For some, this came down to fortuitous triggers:

*‘There was a patient in the next bed to me, she worked for the Probation Service and she was in [hospital] poor lass, a lovely girl, and she said you are entitled to some money, she said get in touch with this …’ (PCP4)*

*‘Yes, we’d heard about Citizens Advice on the telly. Because we are not up on things like those, you know.’ (PCC2)*

For others, a friend or relation with relevant expertise provided a route to support.

*‘So [my daughter] came and she was helping us, cos she knows a lot, because she works for social services.’ (PCC4)*

One participant was treasurer for a peer support group and accessed SWL information through guest speakers.

##### Professional support

Most participants who accessed SWL support described being largely dependent on a professional from any background asking the right questions. It was clear that professionals had a critical role in identifying SWL issues and providing, or signposting to, the necessary support.

*‘And if it wasn’t for X [Council benefits advisor], I just would have been none the wiser. We would have been stuck in financial hell and I daren’t think what that would have meant for me health.’ (HP11)*

*‘So I had to fill some forms in, well actually I filled them all in wrong, so the doctor said to take it to the Citizens Advice Bureau and they’ve got specialist people there to help.’ (PCP2)*

Willingness of professionals to take full responsibility, including form filling and advocating, was gratefully received.

*‘I didn’t know nothing, it was all done. As I say the Macmillan Nurse did all the work and I knew nothing until it was going into me bank account.’ (HP7)*

*‘[The specialist palliative care nurse] said “You’ll have your mobility sticker for the car?” and I said “They knocked us back” and she says “You what? It will be through the door within two days” and it was.’ (PCP3)*

#### Theme 4: Making plans for future incapacity and death

Interviews revealed three main elements to planning for the future: thinking about choices and wishes for the future, talking to family, friends, and professionals about those wishes, and acting on those wishes either in relation to formal documentation, or verbal agreement with family or professionals. Whilst this process might intuitively seem linear, interviews showed that thinking, talking and taking action could occur in any order and potentially without traversing all three. Some stayed firmly at the thinking stage, while others moved straight to acting on their wishes without talking about their wishes with the people who would become their advocates. These stages of planning for the future were often described in the context of challenges and enablers.

#### Theme 5: Challenges to planning for incapacity and death

##### Facing the end of life

Looking ahead felt too challenging for some participants. This proved a barrier to both thinking about the future and open discussion about wishes.

*‘I should really think about my funeral and I’ve got a friend on standby when I need to do that… but I’m not ready for that yet, well I know I’m not, I’m not ready for that stage yet. I don’t want to go there in my mind yet.’ (HP11)*

*‘But... I keep thinking, putting it off, it is silly really but I don’t know it seems a bit final you know, when you’re doing that sort of thing.’ (PCP4)*

For some participants, open discussion represented an acknowledgement of poor prognosis which they felt unable to face. Even some who had completed wills or LPAs struggled with open conversations, risking inadequate information for people with responsibility for decisions in the future.

*‘It did make me [realise that she won’t get better] but not her. She is still is in the hope that, you know, there will be something they could do for her you know to get better (tearful).’ (HC1).*

*‘It’s never been a subject we have talked about other than that she said she wants to be cremated.’ (HC3; appointed attorney: Health and Welfare, Property and Finance)*

##### Cost

For some participants who had progressed to open conversations about the future, cost proved a barrier to taking action. Some had researched costs and felt these were prohibitive, others made assumptions and didn’t investigate further. Several participants worried that costs could escalate and run out of control, and concluded that it was better to simply do without.

*‘It was going to cost £200 each for a will, just to say that if I die first my husband gets everything.’ (HP9)*

*‘We just thought … it [LPA] would have cost too much… So we just left it.’ (HP1)*

*‘I wouldn’t know how to get Power of Attorney, but that costs doesn’t it? I wouldn't want to pay for it.’ (PCC4)*

##### Process

Some participants attempted to formalise plans for the future, including organising wills, LPA or funeral planning, but described getting stuck on a procedural aspect with the result that the process was never finalised.

*‘I organised for a will …Someone sent all the paperwork out but …I still haven’t got it signed … Well it’s not me that has to sign it, it’s the witnesses…It’s daft really but it’s just lining everything up isn’t it?’ (HP7).*

*‘I went into the bank and explained what I wanted doing and she said my son and daughter would have to come to sign the forms, but it’s getting them both together to sign the form and I couldn’t get them both together… so it’s never happened, so I don’t know what to do there.’ (PCP2)*

One participant identified the difficulty engaging with planning ahead when current needs (in this case, heating the home) were unmet.

*‘Anyway, my worry isn’t about planning for tomorrow. I need help now.’ (PCC4)*

#### Theme 6: Enablers to planning for incapacity and death

##### Professional support

Professional support was identified by many participants as the key enabler to planning ahead, in particular, healthcare professionals who encouraged thinking and talking about the future. Although many participants acknowledged that future choices and plans were something they had thought about, having options clarified empowered participants to make informed choices.

*‘we were recommended by her [Parkinson’s Nurse] to deal with those things, putting in place the Power of Attorney, proper guardian and to make reciprocal wills.’ (SOC1)*

Many participants highlighted the need for professionals to initiate discussion around planning for the future, acknowledging that this was a difficult subject to raise themselves.

*‘Yeah actually I know the staff now so I would feel ok about it if they starting talking about the what ifs but I wouldn’t … yah nah, I wouldn’t bring it up.’ (HP6)*

*‘Someone just giving us a big push to do that, oh I’d do it [plan for future], I’d definitely do it.’ (PCP1)*

##### Personal experience

Prior experience of supporting family, friends or neighbours with life-limiting illness empowered some participants to navigate future planning to completion.

*‘..she had [Legal] Guardianship for her mother … We already knew about it so … we knew what it involved.’ (HC1)*

Confidence in a professional, through prior or personal experience, proved an important enabler to formalising plans. This was described as making difficult conversations more comfortable and trust proved important in formalising plans according to individual wishes.

*‘I wanted to make sure that we had our wills authorised properly once I was diagnosed. It was through a solicitor. It’s through, as it happens, the son of a very good friend of ours who is a solicitor.’ (HP5)*

*‘We asked him [friend who is a funeral director] to come out. It was someone I trust and that goes a long way.’ (PCP3)*

#### Theme 7: Timing of SWL support

Some participants identified the challenge of emerging legal needs, especially relating to employment, before a diagnosis was reached. This proved an additional burden at a time they were already facing illness and uncertainty.

*‘I was … struggling, I really did, you know I was giving up jobs and… just drowning really.’ (HP11)*

*‘…they’ve called us a hypochondriac saying I’m trying to get out of late finishes.’ (HP6)*

Participants articulated the need for proactive legal support, early in the illness journey.

*‘… a person like [welfare rights officer] at the [Motor Neurone Disease] Centre, ought to be available everywhere. So that somebody could actually come and see a person who’s on the beginning of the journey. Not at the end of the journey, but at the beginning of the journey.’ (PCC6)*

*‘At the very start, so that you’re not worried about how you’re going to pay your mortgage, how you’re going to do this, how you’re going to do that.’ (HP9)*

There was recognition also that the timing of planning ahead was important, in order to enable people to formalise their wishes:

*‘we should get it [will] done really before he gets any worse and he can’t sign now, he can’t write or anything. So I don’t know how you go on with that.’ (HC4)*

#### Theme 8: Impact of meeting legal needs

Managing finances and benefit entitlements generated an overwhelming sense of relief for many participants.

*‘So it’s little things like that which have made a massive difference to our lives. It has taken the stress off so we can focus on living our lives. … Don’t get me wrong … it’s not about the money. It’s [that] I can’t put a price on the peace of mind that brings.’ (HP11)*

*‘Why it’s just you’ve got no worries have you? You feel like you’re sort of in a cocoon, you know that you can’t get any bother, coz you’ve got everything what you require and you can live life to the full’ (PCP4)*

Even modest financial gain was reported as making a significant difference to perceived quality of life, allowing people to attend to their basic needs, such as heating their home.

*‘My god I nearly fainted, I got over £1000 and thought I’ll be able to get all sorts with this…’ (PCP2).*

Some participants who had made plans for the future, including wills, Power of Attorney and funeral pre-payment, were pleased they had taken control and relieved family members of additional stress at a difficult time.

*It did make me feel very content that things [will and LPA] were in place. (HC2)*

*‘They [children] wouldn’t cope…I don’t want them to worry and chew on and stress … so my funeral’s all sorted’ (PCP2)*

*‘I like being organised and knowing that the family will be provided for.’ (HP5)*

## Discussion

### Main findings

Everyday legal needs are common in chronic illness [3]; our study suggests legal needs, relating to matters of daily life and planning ahead, are prevalent in life-limiting illness. These impact on patients and main carers with both groups in this study describing significant psychological and practical consequences from active needs.

There is no clear route to support for SWL needs and no consistency in practice. Responses from participants revealed a recognition of unmet needs but uncertainty about rights and entitlements or how to access help. A mixture of frustration, bewilderment and a battle mentality was evident when trying to find support for themselves.

All participants had received some support for legal needs, accessed from a range of professions and organisations across health, council, charitable, legal and advice sectors. Routes to support were highly variable. Prior personal experience or fortuitous triggers facilitated access to support, but most participants were guided by informed and engaged professionals. Healthcare professionals, from a variety of roles, proved to be ‘critical noticers’ [3] of SWL needs; identifying them, directly managing them or signposting on to other services. Healthcare professionals have the unique opportunity, as trusted intermediaries, to link people to expertise beyond clinical services, as part of a holistic approach to care. Whilst our study demonstrates the importance of supportive professionals, it raises the concern that many healthcare professionals overlook, or fail to recognise a role in supporting SWL issues [3]. Failure to link health with social welfare risks a spiral of ill health, increased health service utilisation and missed opportunities to enact people’s rights [3].

The impact of meeting legal needs was clear, with participants describing improvements in wellbeing and daily life. Participants were unanimously grateful for the support they received. This was particularly evident when professionals had essentially taken control of resolving SWL issues with minimal demand on patients or carers. Small interventions, such as organising a mobility sticker or access to a benefit, made a big difference.

Timing of SWL support was important, with problems relating to employment predating diagnosis in some cases, and significant psychological and practical burden resulting from delays to accessing SWL advice and support. This reinforces concerns that lack of awareness and poorly defined routes to support result in significant unmet need [3, 5, 6].

Discussion around planning for the future generated some important insights. Participants described three phases of planning ahead: thinking, talking and acting. Although all three seem necessary for effective planning, these proved non-linear and participants engaged with some, but not necessarily all, elements. For some, setting affairs in order generated a sense of control and reduced a perceived burden for family, supporting findings elsewhere [19, 24]. For many, an additional dimension to meeting legal needs was revealed. This related to the challenge of facing a limited prognosis and planning for incapacity and death. Many participants struggled with this, with a perceived focus on loss, and this limited open discussion about choices with family, even a nominated decision-making attorney. This may explain the outstanding unmet needs in the hospice participants and the desire of almost half of participants to leave some aspects of future planning unsorted.

Some participants attempted to formalise plans but encountered difficulty with the process. Our findings reinforce other studies in identifying a range of barriers to engagement with planning in advance, including lack of awareness of the opportunity, limited understanding of the options, lack of support to make decisions around care choices [24, 25] and difficulty anticipating the future [19].

Again, healthcare professionals were identified as key enablers to planning, particularly supporting thinking and informed discussion. Difficulty facing the future was, for some, mitigated by compassionate and supportive professionals.

The majority of participants had unresolved everyday legal needs, identified through interview questions and discussion; most, but not all, wanted help resolving at least some of these. For hospice participants, unresolved issues related to planning for the future; primary care recruits had broader needs. This may reflect access to specialist social workers in the hospice but more fragmented services in the community setting. Aside from this, there was little difference evident between experience of participants from the two recruitment sites, or between patients and carers.

Whilst individual autonomy in decision making is promoted as a basic human right and civil law defines rights, entitlements and protections in daily life, this study reveals inconsistent approaches to delivery of these rights with resulting adverse impacts on patients and carers. Key barriers relate to lack of awareness of legal issues and the law as a route to resolution and lack of clear routes to support. These barriers restrict self-help as well as professional engagement.

### Implications for practice

This study raises the need, and opportunity, for early and effective management of everyday legal issues. It also highlights the central role of healthcare professionals in recognising everyday legal needs and enabling support, either directly or signposting on to appropriate agencies. Improving care necessitates increased awareness of legal issues and associated rights, amongst professionals as well as people living with life-limiting illness. Manageable information for the public would enable self-help. An accessible, integrated pathway of care across the breadth of relevant agencies is required to manage SWL needs in life-limiting illness. This needs to be inclusive, irrespective of diagnosis and place of care. Our study has identified that this needs to be proactive and available early in the disease journey, at a time when legal needs, such as employment issues, are developing. Navigation across agencies is likely to be facilitated by social prescribing link workers [26].

This aligns with the whole system approach described in the Comprehensive Model for Personalised Care [27] and proposals around access to care and support to promote greater independence and autonomy in daily life [28]. Further work by the research team includes interprofessional education, to raise awareness and confidence around legal needs and rights in life-limiting illness, and the coproduction of care pathways. Education on human rights in the context of end of life care has been developed and delivered by Sue Ryder. Evaluation of this programme has shown the need and impact of a rights-based approach to care [29].

Alongside SWL expertise, an interdisciplinary team needs to offer confident communication skills to support sensitive conversations around the challenge of facing the future and enable people to assert their choices and rights. There is a strong focus on advance care planning within end of life policy [1, 2] but this study, and others, highlights barriers to engagement with this process. Earlier recognition that people might be approaching end of life, coupled with a stronger focus on living well and meeting the full range of needs, could be helpful [19]. Refocussing planning for the future, from discussion around loss to empowerment and defining wishes, could support effective engagement. In this context, individual autonomy could be reframed as relational autonomy; independence as interdependence [19], thus generating an enabling partnership approach between patient, family and professionals in which rights and personal priorities are heard and options negotiated. At national strategy level, an approach to sustainable and accessible health-justice partnerships is under consideration [14].

### Limitations

Legal needs do not have an agreed definition; we adopted a working definition based on findings of previous scoping work [11]. One of our aims is to increase understanding of everyday legal needs; hence it was important to quantify the type and frequency of legal needs in our analysis. This study was not designed to provide a powered quantitative analysis and therefore definitive conclusions cannot be drawn from this. However, it provides an important starting point for dialogue with policy makers and commissioning bodies about the significance of legal needs.

Configuration of palliative care and advice services shows significant geographical variation. Our findings based on two sites within one region, will, to some extent, reflect local service provision. This may limit the generalisability of findings. However, our findings do reflect the opinions of national stakeholder organisations [11] and are aligned with evidence relating to chronic illness [3–6].

Interviews and data analysis were conducted by professionals with clinical experience and prior knowledge of the subject under investigation. We recognise that this will have affected our interaction with the data. We used open questions in the early stage of the interview, determined with the help of patient/carer representatives within the steering group, to allow participants to share experience freely. As part of our data analysis, we agreed and refined themes through in-depth discussion and cross-checking across transcripts to bring different perspectives on the same data. Participants often felt strongly about their experiences, generating repeated themes which had implications for services and practice. This, coupled with our previous work on end-of-life care, allows us feel confident that we have identified and presented themes that are representative of the issues that were important to participants and relevant to service delivery.

Dyads generated variation in interview techniques, with hospice dyads interviewed separately, but primary care dyads requesting to be interviewed together. This did not reflect our intended methodology but the sensitivity of the discussion required appropriate flexibility. The interviewer was careful to capture views of both participants but we recognise the potential for bias inherent in joint interviews.

## Conclusions

Everyday legal needs commonly affect people living towards end of life, and their informal carers, and can create significant challenges. Our study has shown that limited awareness of the issues and individual rights, together with lack of clear routes to support, generates frustration, variability in practice and unmet needs; resolution of legal needs results in practical and emotional benefit. Healthcare professionals hold key roles as ‘critical noticers’, trusted intermediaries and compassionate enablers, resolving legal needs directly, referring onto other agencies and facilitating difficult conversations around planning. Everyday legal needs must be included within holistic care towards end of life, integrating and utilising the full breadth of available services and expertise.

## Data Availability

The datasets used and analysed during the current study are available from the corresponding author on reasonable request.

## Abbreviations

SWL: Social welfare legal
GP: General Practitioner
REC: Research Ethics Committee
